# Protective Effects of Resveratrol on Heat Stress‐Induced Liver Dysfunction in Broilers Through Modulating Autophagy and NLRP3 Inflammasome

**DOI:** 10.1002/fsn3.71326

**Published:** 2025-12-05

**Authors:** Kang‐Ning Ding, Zi‐Hao Li, Jia‐Ci Cai, Hui‐Lin Li, Yang Yang, Ya‐Qiong Ye, Lu‐Ping Tang

**Affiliations:** ^1^ School of Animal Science and Technology Foshan University Foshan China

**Keywords:** autophagy, heat stress, liver, NLRP3, resveratrol

## Abstract

This study aims to explore the effects of resveratrol on the NLRP3 inflammasome and its regulatory mechanism in the liver of heat‐stressed broilers. Broilers were randomly divided into three groups: (1) Control (23°C ± 2°C), (2) Heat stress (HS, 35°C ± 2°C, 8 h/day), and (3) Resveratrol (HS + resveratrol) group. Resveratrol supplementation (400 mg/kg diet) commenced 48 h prior to HS and continued throughout the experimental period. The results showed that resveratrol improved growth performance with a higher average daily gain (ADG) and lower feed conversion ratio (FCR), and increased liver weight and index along with reduced inflammatory cell infiltration in broilers. In addition, resveratrol upregulated the autophagy‐related genes and protein levels in the liver. Moreover, resveratrol obviously down‐regulated the protein and mRNA levels of the NOD‐like receptor protein 3 (NLRP3) inflammasome. In conclusion, resveratrol mitigated heat stress‐induced liver injury by modulating autophagy and down‐regulating NLRP3 inflammasome activation.

## Graphical abstract

Resveratrol improved growth performance and alleviated liver injury in heat‐stressed broilers. It restored autophagy by regulating the mTOR‐ULK1 signaling pathway. It also inhibited NLRP3 inflammasome activation, revealing a protective autophagy–NLRP3 regulatory mechanism under heat stress. The study offers new mechanistic insight and a promising nutritional strategy for improving broiler resilience in hot climates.

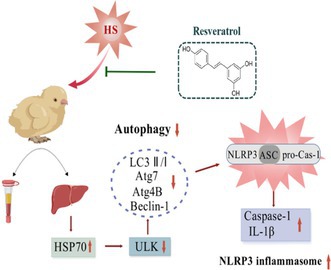

## 1 Introduction

Heat stress (HS) can impair production performance and restrict the development of the poultry industry. As of 2003, HS caused economic losses exceeding 128 million dollars annually in the United States poultry industry (St‐Pierre et al. 2003). With the intensification of global warming, these losses are expected to rise in the coming years (Chen et al. 2021). Veterinary clinical studies indicate that HS exacerbates oxidative stress and inflammation in the liver, which is a key factor contributing to decreased growth performance in poultry (Chen et al. 2021; Park et al. 2022). Our previous study confirmed that HS‐induced growth performance impairment is directly related to liver damage (Tang et al. 2022). However, the precise pathological mechanisms underlying HS‐induced liver injury in poultry remain unclear, limiting the development of effective therapeutic strategies.

The NLRP3 inflammasome is a critical regulator of the inflammatory response. Upon stimulation by exogenous or endogenous danger signals, NLRP3 recruits the downstream apoptosis‐associated speck‐like protein ASC, initiating its assembly and activating caspase‐1 (Kodi et al. 2024). Activated caspase‐1 promotes the release of pro‐inflammatory cytokines IL‐1β and IL‐18, thereby exacerbating tissue inflammatory response and damage (Zhou et al. 2011). Studies have shown that modulation of the NLRP3 inflammasome can alleviate hepatic inflammation in broilers; for example, selenomethionine significantly inhibits the NF‐κB/NLRP3 signaling pathway to reduce lipopolysaccharide‐induced hepatic injury in broilers (Qu et al. 2020). Additionally, *Macleaya cordata* extract reduces hepatocellular pyroptosis by downregulating NLRP3 inflammasome‐related factors, thereby improving growth performance in broilers (Liu et al. 2022). These studies suggest that NLRP3 inflammasome activation may be a central mechanism underlying HS‐induced hepatic injury in poultry. However, the upstream regulatory mechanisms controlling NLRP3 activation under HS remain largely unexplored, representing a critical knowledge gap.

Autophagy is a key cellular homeostatic process that negatively regulates NLRP3 inflammasome activation. Inhibition of autophagy has been shown to promote NLRP3 inflammasome activation through multiple triggers, including ATP, nigericin, and lysosomal disruption. Previous studies reported that HS (35°C for 1 week) induced liver injury in broilers, closely associated with oxidative damage (Wen et al. 2021). Our previous work demonstrated that HS downregulates the autophagy marker LC3 in the liver, suggesting impaired autophagy contributes to hepatic damage in broilers (Tang et al. 2022). However, the functional link between autophagy impairment and NLRP3 inflammasome activation under HS in poultry remains unclear. Understanding this autophagy–NLRP3 axis is critical, as it may provide a mechanistic basis for targeted interventions to protect liver function under HS conditions.

Resveratrol is a natural polyphenolic compound widely found in grapes, peanuts, berries, and red wine, has attracted considerable attention due to its potent antioxidant, anti‐inflammatory, and hepatoprotective effects (Tejada et al. 2021). In poultry nutrition, resveratrol has emerged as a promising agent to enhance stress resistance and organ health (Meng et al. 2023). Nevertheless, the potential of resveratrol to modulate the autophagy–NLRP3 axis under HS in broilers has not been investigated. Therefore, this study aims to explore whether resveratrol alleviates via regulation of autophagy and suppression of NLRP3 inflammasome activation, providing both mechanistic insight and practical relevance for poultry production.

## 2 Materials and Methods

### 2.1 Animals and Group

Ninety two‐week‐old broilers were provided by the specific pathogen free (SPF) experimental animal center at Wenshi Xin xing dahuanong eggs Co. Ltd. (Guangdong, China). Broilers were housed in a controlled environmental chamber with temperature and humidity conditions maintained at 23°C ± 2°C and 70% ± 10%, respectively. Following a 1 week acclimation, the broilers were randomly assigned into 3 groups: control, heat stress (HS) and resveratrol (HS + resveratrol), with 30 replicates in each group. Broilers in the HS group were exposed to HS conditions (35°C ± 2°C, 70% humidity) for 8 h/d (8:00–16:00) for 7 consecutive days. For the remaining 16 h of the day, the broilers were maintained at standard (23°C ± 2°C, 70% humidity), identical to the environment of the control group. A resveratrol‐enriched diet (400 mg/kg) (Zhang et al. 2023) was administered for 2 days prior to HS, continuing for 9 days. Body weight and feed consumption were recorded daily throughout the experiment. After 1 week of HS, blood samples were collected via jugular vein for the assessment of liver function biomarkers. Following blood collection, broilers were euthanized using a pentobarbital sodium injection. Liver tissues were collected and either fixed in 4% paraformaldehyde for histological examination or stored at −80°C for subsequent gene and protein expression analyses.

The broilers' feed was supplied by WENS FOODSTUFF GROUP CO. LTD, and consisted of wheat, corn, soybean oil, soybean meal, along with other conventional feed additives, vitamins, trace elements, and amino acids. Resveratrol (≥ 99%, HPLC grade) was obtained from Sigma‐Aldrich (Steinheim, Germany).

### 2.2 Growth Performance Analysis

To calculate the average daily gain (ADG), average daily feed intake (ADFI), and feed conversion ratio (FCR), feed intake and body weight of each broiler were recorded from 21 to 35 days of age. The FCR was calculated using the formula: FCR = ADFI/ADG.

### 2.3 Histological Changes and Liver Index Analysis

The entire liver was taken from each broiler and weighed. The liver index was calculated using the formula: Liver index = liver weight (g)/body weight (g) × 100%.

Liver tissues were fixed in 4% paraformaldehyde solution for 48 h, processed into paraffin blocks, and sectioned into 5 μm slices. The sections were stained with hematoxylin and eosin (H&E) and examined under an optical microscope (Bio‐Rad, USA) at 400 × magnification to assess histological changes.

### 2.4 Determination of Serum Liver Function Indices

Serum levels of alanine aminotransferase (ALT), aspartate aminotransferase (AST), total protein (TP), alkaline phosphatase (ALP) and albumin (ALB) were measured using the Alanine Aminotransferase Assay Kit (105‐000442‐00), Aspartate Aminotransferase Assay Kit (105‐000443‐00), Total Protein Assay Kit (105‐000451‐00), Alkaline Phosphatase Assay Kit (105‐000444‐00) and Albumin Assay Kit (105‐000450‐00). Direct bilirubin (D‐Bil) and total bilirubin (T‐Bil) content were quantified using the Direct Bilirubin Assay Kit (105‐000455‐00) and Total Bilirubin Assay Kit (105‐000454‐00). All kits were obtained from Mindray Bio‐Medical Electronics Co. Ltd. (Shenzhen, China).

### 2.5 Western Blot Analysis

Total protein was extracted from liver tissues using RIPA lysis buffer on ice. Protein concentration was quantified using the BCA protein quantification kit (ThermoFisher Scientific, USA). Protein extracts were separated by SDS‐PAGE electrophoresis and transferred to a polyvinylidene difluoride (PVDF) membrane. Membranes were blocked with 3% BSA for 1 h and then incubated overnight at 4°C with diluted primary antibodies (HSP70, ULK1, Beclin‐1, Atg7, Atg4B, LC3B, NLRP3, Caspase‐1, IL‐1β and GAPDH; Cell Signaling Technology, Danvers, MA, USA) diluted in 3% BSA. Then, membranes were further incubated with secondary antibodies at 37°C for 1 h. The protein bands were visualized using the ECL Kit (Beyotime, China) and a chemiluminescent imaging system (Tanon 5200, Shanghai, China). Densitometric analysis of the bands was performed using Image J software. Each experimental group consisted of six biological replicates, and each sample was analyzed twice (technical replicates).

### 2.6 Immunofluorescence Analysis

After deparaffinization with xylene and rehydration through a graded alcohol series, tissue sections were subjected to antigen retrieval by boiling in citrate buffer. The cell membranes were permeabilized using 0.1% Triton X‐100, and endogenous peroxidase activity was quenched by incubating the slides with 3% H_2_O_2_ at 37°C for 10 min in the dark. Then, the sections were incubated with primary antibodies (NLRP3 and LC3B) from different species at 4°C for 12 h. The slides were incubated with fluorescent‐labeled secondary antibodies for 1 h in the dark. Images were captured using a fluorescent microscope (IX53, OLYMPUS, Japan). Each group included four biological replicates, and each sample was tested in triplicate (technical replicates).

### 2.7 Quantitative Real‐Time Polymerase Chain Reaction (qRT‐PCR)

Total RNA was extracted from liver tissues using Trizol Reagent (Hlingene Corporation, Shanghai, China) and reverse transcribed using the 1st Strand cDNA Synthesis kit (Takara, Tokyo, Japan). Following cDNA synthesis, qRT‐PCR was performed with the TB Green Premix Ex Taq II (Takara, Tokyo, Japan) using a Roche Light‐Cycler 480 system (Roche, Switzerland). The relative fold changes in gene expression were calculated using the 2^−ΔΔCt^ method. The gene primers used for qRT‐PCR are listed in Table 1. Each group included four biological replicates, and each sample was analyzed in triplicate (technical replicates).

**TABLE 1:** The primers in qRT‐PCR.

Gene	GenBank	Primer sequences (5′‐3′)	Produce size
mTOR	NM_020009	F:GAAGAGCTGATTCGGGTAG R:ACCATTCTTGTGCCTCCATT	214 bp
Beclin1	NM_001006332	F:CGTATGGCAACCACTCGTATT R:TTATTGTCCCAGAAGAACCTCAG	97 bp
LC3I	NM_001177415	F:TTACACCCATATCAGATTCTTG R:ATTCCAACCTGTCCCTCA	143 bp
LC3II	NM_001031461	F:AGTGAAGTGTAGCAGGATGA R:AAGCCTTGTGAACGAGAT	193 bp
NLRP3	NM_001348947	F:GCTCCTTGCGTGCTCTAAGACC R:TTGTGCTTCCAGATGCCGTCAG	150 bp
Caspase‐1	XM_040687588	F:CAAGAGTAATGGGACCACGGACATC R:CACGGCAGCACTGGATAATGACC	119 bp
IL‐1β	NM_204524	F:CACTGGGCATCAAGGGCTACAAG R:GTCCAGGCGGTAGAAGATGAAGC	140 bp
β‐Actin	NM_205518	F:ACGTCTCACTGGATTTCGAGCAGG R:TGCATCCTGTCAGCAATGCCAG	298 bp

Abbreviations: Caspase‐1, Cysteinyl aspartate‐specific proteinase 1; LC3, Full name MAP1LC3 (microtubule‐associated protein light chain 3); NLRP3, NOD‐like receptor protein 3.

### 2.8 Statistical Analysis

Data were presented as the means ± standard deviation (SD). All statistical analyses were performed using SPSS 17.0 software (SPSS, Chicago, USA). The data were analyzed by one‐way analysis of variance (ANOVA) followed by the LSD post hoc test. Differences were considered statistically significant at *p <* 0.05.

## 3 Results

### 3.1 Resveratrol Enhanced Growth Performance of Broilers Exposure With Heat Stress

Compared to the control group (Table 2), the body weight, ADFI and ADG of broilers in the heat stress group were decreased (*p <* 0.05, *p* < 0.01, *p* < 0.01, respectively). The FCR in heat‐stressed broilers was significantly higher than that in the control group (*p <* 0.05). There were no significant differences in weight gain and ADFI between the HS and resveratrol groups. However, resveratrol ameliorated growth performance in heat‐stressed broilers, resulting in higher ADG and lower FCR (*p* < 0.01, *p* < 0.05, respectively).

**TABLE 2:** Effects of resveratrol on growth performance of broilers exposed to heat stress.

	Control	Heat stress	Resveratrol
Weight gain (g)	86.92 ± 10.47	62.64 ± 7.10^#^	63.06 ± 8.50
ADFI (g/d)	39.37 ± 5.70	30.59 ± 3.76^##^	30.75 ± 3.09
ADG (g/d)	15.73 ± 1.77	7.63 ± 1.14^##^	11.42 ± 2.40**
FCR (%)	2.57 ± 1.07	4.02 ± 1.05^#^	2.65 ± 1.38*

*Note:* ^#^ *p* < 0.05, ^##^ *p* < 0.01 vs. control group; *p < 0.05, **p < 0.01 vs. heat stress group (Applicable for full text). (*n* = 30).

Abbreviations: ADFI, average daily feed intake; ADG, average daily gain; FCR, feed conversion ratio.

### 3.2 Resveratrol Ameliorated Heat Stress‐Induced Liver Inflammatory Infiltration

**FIGURE 1:**
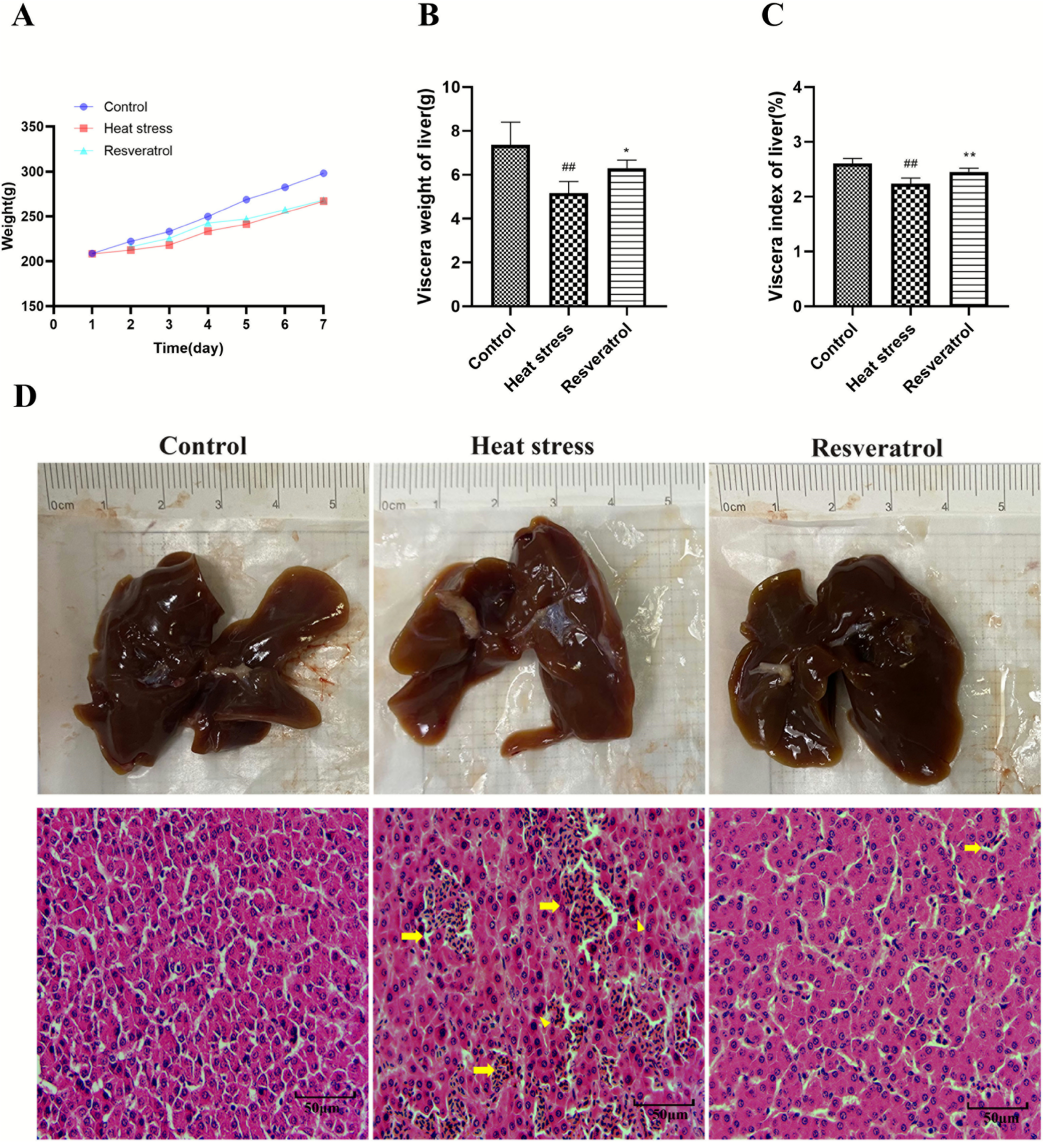
Effects of resveratrol on liver changes in broilers exposed to heat stress. (A) body weight, (B) viscera weight, and (C) liver index in broilers were measured (*n* = 30). (D) Histopathological changes in the liver (H&E staining) were observed (*n* = 3). Yellow arrows indicate heterophil infiltration, and the yellow triangle represents neutrophil infiltration. ##*p* < 0.01 vs. Control group; *p < 0.05, **p < 0.01 vs. Heat stress group.

The data regarding liver changes were presented in Figure 1. Compared with the control group, heat stress for 1 week significantly reduced liver weight and index in broilers (*p <* 0.01, *p <* 0.01, respectively). However, resveratrol improved these two parameters (*p <* 0.05, *p <* 0.01). After necropsy (Figure 1D), the liver surface texture appeared normal in all 3 groups of broilers, with no obvious necrotic points observed. Liver histological examination revealed inflammatory infiltration (*neutrophils and lymphocytes*) and *disordered hepatocyte arrangement* in heat‐stressed broilers, while resveratrol ameliorated these signs of inflammation and disorganization. These results suggest that resveratrol enhances growth performance, potentially by mitigating liver tissue damage in heat‐stressed broilers.

### 3.3 Resveratrol Ameliorated Heat Stress‐Induced Liver Dysfunction of Broilers

As shown in Table 3, compared with the control group, heat stress significantly increased serum AST level (*p <* 0.01), while TP, D‐Bil and ALB II levels were decreased (*p <* 0.01, *p <* 0.01, *p <* 0.05, respectively). In the resveratrol group, AST levels were lower than those in the heat stress group (*p <* 0.01), while TP and ALB II levels were higher (*p <* 0.05, *p <* 0.05, respectively). These results suggest that resveratrol inhibited the deterioration of serum hepatic function indices induced by heat stress.

**TABLE 3:** Effects of resveratrol on serum liver function indices in broilers exposed to heat stress.

Group	Control	Heat stress	Resveratrol
ALT (U/L)	3.64 ± 0.43	3.34 ± 0.87	3.55 ± 0.63
AST (U/L)	245.77 ± 10.12	283.56 ± 29.49^##^	259.09 ± 9.27**
TP (g/L)	30.41 ± 0.96	27.83 ± 0.59^##^	29.91 ± 0.44*
ALP (U/L)	1948.01 ± 336.95	2782.54 ± 648.73	2093.70 ± 222.53
D‐Bil (μmol/L)	8.45 ± 0.79	7.77 ± 0.49^#^	7.80 ± 0.74
T‐Bil (μmol/L)	39.01 ± 0.44	41.18 ± 0.28	39.94 ± 0.58
ALB II (g/L)	11.49 ± 0.43	10.71 ± 0.43^#^	11.33 ± 0.18*

*Note:* ^#^ *p* < 0.05, ^##^ *p* < 0.01 vs. control group; *p < 0.05, **p < 0.01 vs. heat stress group. Data are presented as least squares means of 3 replicated cages per treatment.

Abbreviations: ALB II, albumin; ALP, alkaline phosphatase; ALT, alanine aminotransferase; AST, aspartate aminotransferase; D‐Bil, direct bilirubin; T‐Bil, total bilirubin; TP, total protein.

### 3.4 Resveratrol Enhanced the Protein Levels of Autophagy‐Related Genes in Broilers Exposure With Heat Stress

**FIGURE 2:**
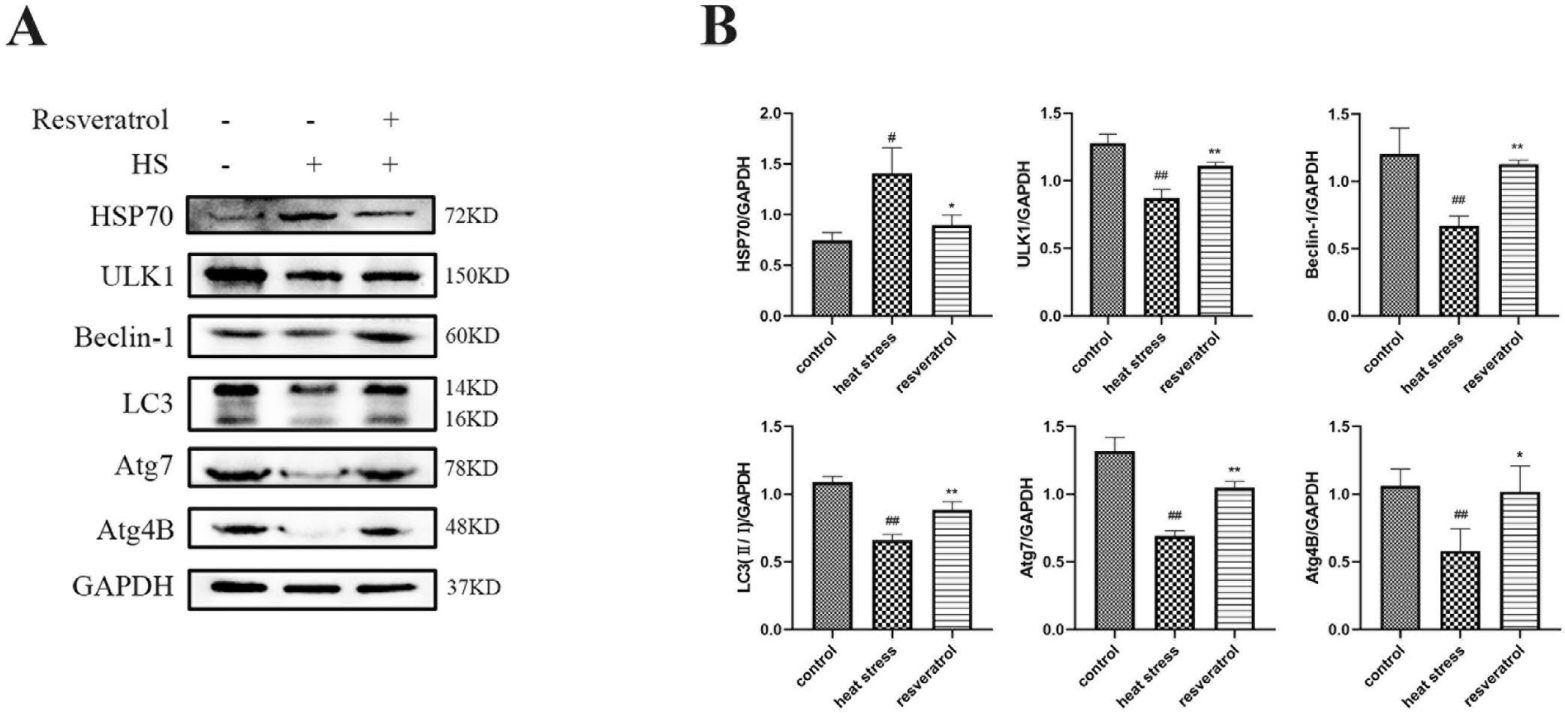
Effects of resveratrol on protein levels of autophagy‐related genes in broilers. #*p* < 0.05, ##*p* < 0.01 vs. Control group; *p < 0.05, **p < 0.01 vs. Heat stress group.

HSP70 plays a critical role in protecting cells from stress‐induced cellular damage. Compared with the control group, HSP70 levels were increased in heat‐stressed broilers (*p <* 0.05, Figure 2). Heat stress also significantly reduced the protein levels of ULK1, Beclin‐1, Atg7 and Atg4B (*p <* 0.01, *p <* 0.01, *p <* 0.01, *p <* 0.01, respectively). These proteins are crucial for the initiation and progression of autophagy, and heat stress impaired the autophagy process in broilers. The LC3II/I ratio, a marker of autophagic flux, was lower in the HS group than in the control group (*p <* 0.05). Resveratrol significantly reversed the changes in the expression levels of HSP70 and all these autophagy‐related proteins. These results indicate that resveratrol mitigates heat stress‐induced autophagy impairment.

### 3.5 Resveratrol Strengthened Gene Levels of Autophagy‐Related Genes in Broilers

**FIGURE 3:**
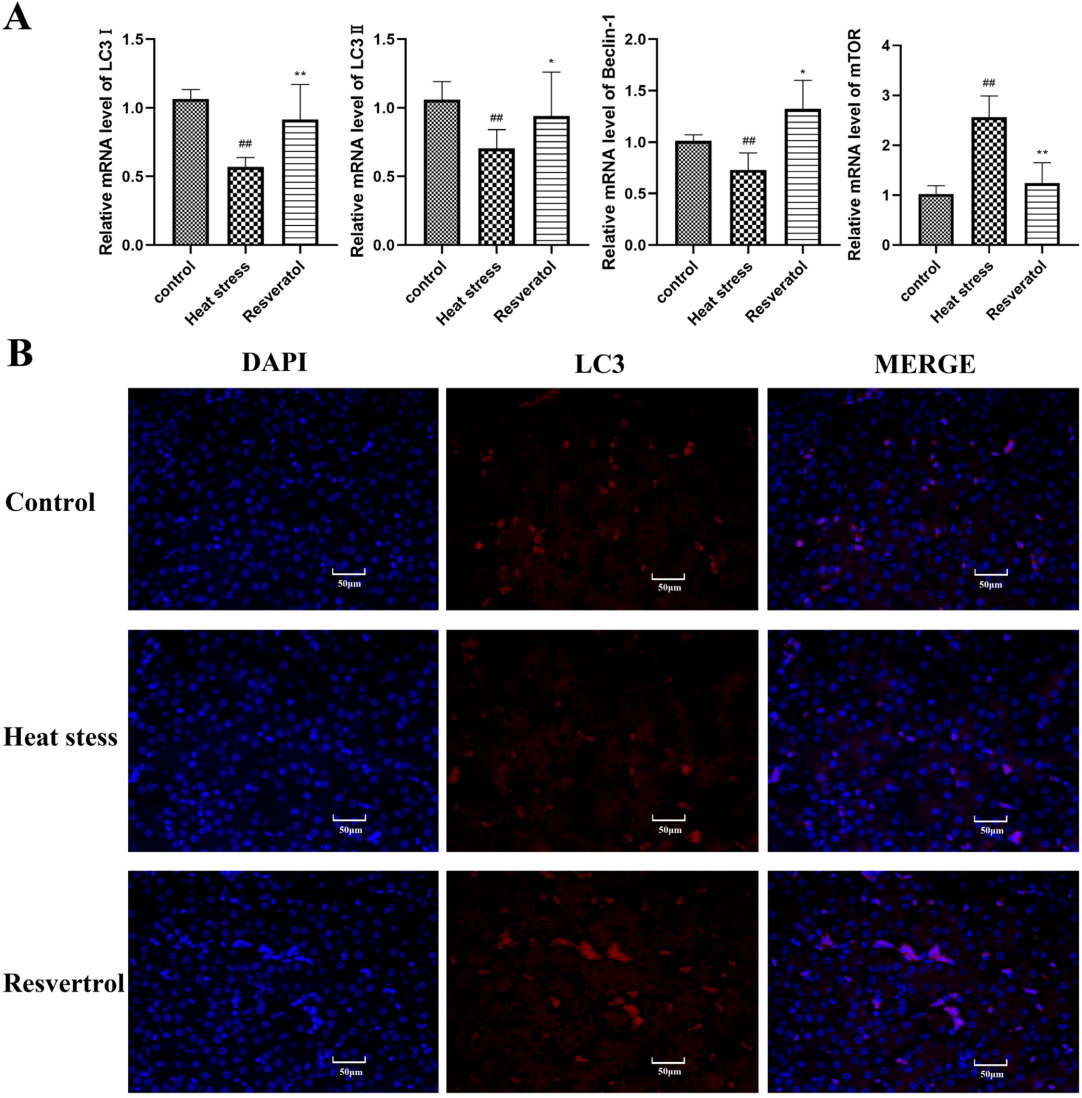
Effects of resveratrol on autophagy levels in broilers. (A) Gene expression levels of LC3I, LC3II, Beclin‐1 and mTOR. (B) LC3B protein levels assessed by immunofluorescence histochemistry. ##*p* < 0.01 vs. Control group; *p < 0.05, **p < 0.01 vs. Heat stress group.

The expression of autophagy‐related genes were assessed by qRT‐PCR (Figure 3A). Heat stress strongly decreased the gene levels of LC3I, LC3II and Beclin‐1 (*p* < 0.01, *p* < 0.01, *p* < 0.01, respectively), while the mTOR gene level was increased (*p* < 0.01). Resveratrol strengthened the gene expression of LC3I, LC3II and Beclin‐1, and reduced the mTOR gene expression in heat‐stressed broilers. Immunofluorescence histochemistry analysis further revealed that LC3B levels in HS group were lower than those in the control group. Broilers in the resveratrol group showed higher LC3B levels in the liver compared to the HS group. These results suggest that resveratrol enhances liver autophagy in broilers exposed to heat stress.

### 3.6 Resveratrol Alleviated NLRP3 Inflammasome Activation in Liver of Heat‐Stressed Broilers

**FIGURE 4:**
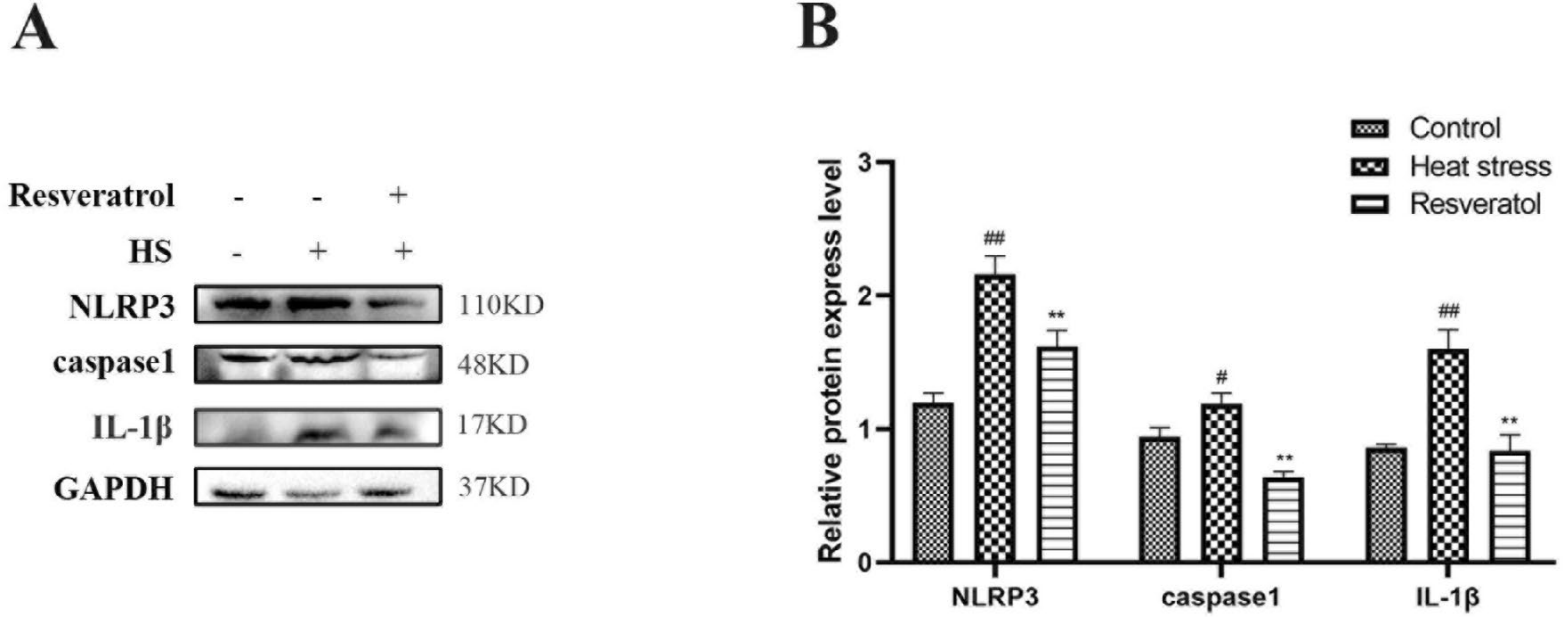
Effects of resveratrol on NLRP3 inflammasome in the liver of broilers. Protein expressions of NLRP3, caspase‐1, and IL‐1β in the liver of broilers were measured by western blotting. #*p* < 0.05, ##*p* < 0.01 vs. Control group; **p < 0.01 vs. Heat stress group.

Heat stress significantly increased the hepatic protein expressions of NLRP3 (*p <* 0.01), caspase‐1 (*p <* 0.05) and IL‐1β (*p <* 0.01), compared to the control group broilers (Figure 4). However, broilers in the resveratrol group exhibited lower levels of NLRP3, caspase‐1 and IL‐1β proteins (*p <* 0.01, *p <* 0.01, *p <* 0.01, respectively) in the liver compared to the HS group. These findings suggest that resveratrol can reduce the activation of the NLRP3 inflammasome in the liver of heat‐stressed broilers.

### 3.7 Resveratrol Decreased the Gene Expression Levels of NLRP3 Inflammasome‐Related Genes in Broilers

**FIGURE 5:**
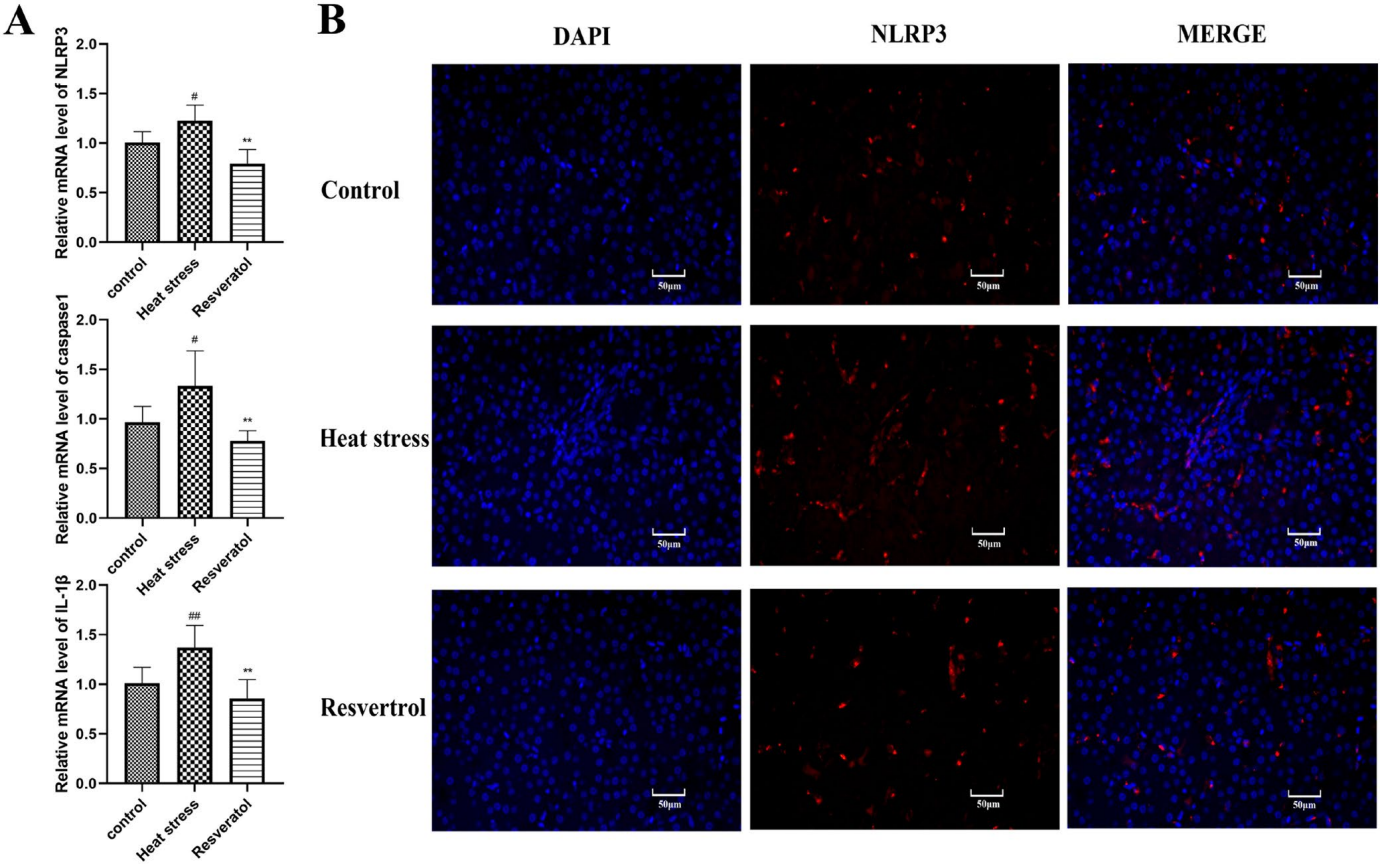
Effects of resveratrol on gene expression levels of NLRP3 inflammasome‐related genes. (A) gene expression levels of NLRP3, caspase‐1 and IL‐1β in broilers. (B) NLRP3 immunofluorescence histochemistry analysis. #*p* < 0.05, ##*p* < 0.01 vs. Control group; **p < 0.01 vs. Heat stress group.

The results in Figure 5A show that the gene expression levels of NLRP3, caspase‐1 and IL‐1β were significantly upregulated in heat‐stressed broilers (*p <* 0.05, *p <* 0.05, *p <* 0.01, respectively) compared with the control group. Immunofluorescence histochemistry analysis confirmed that heat stress increased the NLRP3 protein levels in the liver of broilers exposed to heat stress (Figure 5B). Resveratrol decreased the gene expression levels of NLRP3, caspase‐1 and IL‐1β, compared with the HS group (*p <* 0.01, *p <* 0.01, *p <* 0.01, respectively). Additionally, NLRP3 protein expression was reduced in the liver of resveratrol‐treated heat‐stressed broilers.

## 4 Discussion

Heat stress is a critical environmental factor that disrupts physiological homeostasis, leading to decreased growth rates and production limitations in broilers (Wasti et al. 2020). Our previous study demonstrated that HS significantly reduced the ADG and ADFI levels in broilers (Liu et al. 2022). This study showed that HS further reduced body weight gain, ADG, and ADFI, while increasing the FCR, indicating clear growth performance impairment. Previous research has shown that nutritional interventions can mitigate HS‐induced performance decline in broilers (Hu et al. 2021). In this study, dietary supplementation with resveratrol (400 mg/kg) improved growth performance under HS by increasing ADG and reducing FCR. These results highlight the potential of resveratrol as an effective nutritional strategy to enhance growth performance in heat‐stressed broilers.

The liver is crucial in thermoregulation in avian species, making it particularly susceptible to heat stress (Emami et al. 2020; Oladokun and Adewole 2022). Using proteomics combined with two‐dimensional gel electrophoresis (2DE) and mass spectrometry, the researchers analyzed protein level changes in the liver of broilers with different production performances, 20 days after heat stress at the same age. The study found that HS significantly increases oxidative stress and inflammation‐related proteins in the liver, which in turn impair growth and development in broilers (Park et al. 2022). This study demonstrated that HS for 1 week reduced both liver weight and index of broilers. Histopathological examination further revealed liver inflammation induced by HS, while resveratrol ameliorated these pathological changes. Biochemical analysis showed that HS elevated AST levels by 1.15‐fold and decreased TP, D‐Bil and ALBII levels, indicating impaired liver function. Resveratrol improved AST and ALBII levels, suggesting a protective effect on liver function. These results suggest that resveratrol promotes liver growth and mitigates liver dysfunction in heat‐stressed broilers, though the precise mechanisms underlying these effects require further investigation.

Autophagy is widely involved in diverse physiological and pathological processes. Beclin‐1, an essential autophagy initiation factor, activates autophagy‐related genes (Atgs) to promote autophagosome formation (Sun et al. 2019). LC3, a key autophagy protein, is required for autophagosome assembly, with the cytosolic LC3I form being converted into the membrane‐bound LC3II during autophagy activation (Rao et al. 2024; Yu et al. 2018). In our study, HS significantly impaired autophagy in the liver of broilers. HS reduced Beclin‐1 gene expression, and decreased protein levels of Beclin‐1, Atg4B and Atg7. The LC3‐II/LC3‐I ratio and ULK1 protein levels were also decreased, and immunofluorescence analysis showed reduced LC3 accumulation in the liver. In addition, mRNA levels of LC3‐I and LC3‐II were significantly decreased, indicating impaired autophagic activity under heat stress. Resveratrol supplementation counteracted these effects by increasing both the gene and protein levels of LC3 and enhancing LC3 immunofluorescence in the liver. These findings suggest that resveratrol protects the liver from HS‐induced damage by enhancing autophagy.

Aberrant activation of the NLRP3 inflammasome plays a vital role in promoting inflammation and tissue damage, and is critically involved in defense against pathogen infections and liver inflammatory diseases (Sayaf et al. 2024). In a mouse model of non‐alcoholic steatohepatitis (NASH), impaired mitochondrial autophagy has been shown to trigger liver NLRP3 inflammasome activation (Huang et al. 2022). Autophagy serves as a negative regulator of NLRP3 activation, helping to maintain cellular homeostasis and mitigate inflammatory injury, as demonstrated in DAMP‐induced acute lung injury models (Peng et al. 2021). However, the functional relationship between autophagy and NLRP3 inflammasome activation in broiler liver remains largely unexplored. Our study demonstrated that HS activated the NLRP3 inflammasome by increasing protein levels of NLRP3, caspase‐1 and IL‐1β, along with elevated gene expression of these markers which was inhibited by resveratrol. Resveratrol supplementation inhibited NLRP3 inflammasome activation, suggesting that it may protect the liver by modulating the autophagy–NLRP3 axis under HS conditions.

## 5 Conclusion

Resveratrol alleviates heat stress–induced liver inflammation in broilers by modulating autophagy and suppressing NLRP3 inflammasome activation, and improves the production performance of heat‐stressed poultry. These findings suggest that resveratrol is a promising dietary supplement to enhance the resilience of poultry in hot climates, leading to better growth performance in poultry farming.

## Author Contributions

Conceptualization and funding, Lu‐Ping Tang and Ya‐Qiong Ye; Methodology investigation, and writing, Kang‐Ning Ding and Zi‐Hao Li; Data curation and supervision, Jia‐Ci Cai, Hui‐Lin Li and Yang Yang. All authors have read and agreed to the published version of the manuscript.

## Funding

This project was supported by Guangdong Provincial Department of Science and Technology [2024A1515030170]. National Natural Science Foundation of China [32402930]. Department of Education of Guangdong Province [2024KTSCX208].

## Ethics Statement

All animal experiments were carried out within the guidelines for the care and use of experimental animals established by the Ministry of Science and Technology of the People's Republic of China, and approved by the Laboratory Animal Management Committee of Foshan University. All efforts were made to minimize animal suffering.

## Conflicts of Interest

The authors declare no conflicts of interest.

## Data Availability

All data supporting the findings of this study are included in the article. No additional datasets were generated or analyzed.
